# Hyperdominance in Amazonian forest carbon cycling

**DOI:** 10.1038/ncomms7857

**Published:** 2015-04-28

**Authors:** Sophie Fauset, Michelle O. Johnson, Manuel Gloor, Timothy R. Baker, Abel Monteagudo M., Roel J.W. Brienen, Ted R. Feldpausch, Gabriela Lopez-Gonzalez, Yadvinder Malhi, Hans ter Steege, Nigel C.A. Pitman, Christopher Baraloto, Julien Engel, Pascal Pétronelli, Ana Andrade, José Luís C. Camargo, Susan G.W. Laurance, William F. Laurance, Jerôme Chave, Elodie Allie, Percy Núñez Vargas, John W. Terborgh, Kalle Ruokolainen, Marcos Silveira, Gerardo A. Aymard C., Luzmila Arroyo, Damien Bonal, Hirma Ramirez-Angulo, Alejandro Araujo-Murakami, David Neill, Bruno Hérault, Aurélie Dourdain, Armando Torres-Lezama, Beatriz S. Marimon, Rafael P. Salomão, James A. Comiskey, Maxime Réjou-Méchain, Marisol Toledo, Juan Carlos Licona, Alfredo Alarcón, Adriana Prieto, Agustín Rudas, Peter J. van der Meer, Timothy J. Killeen, Ben-Hur Marimon Junior, Lourens Poorter, Rene G.A. Boot, Basil Stergios, Emilio Vilanova Torre, Flávia R.C. Costa, Carolina Levis, Juliana Schietti, Priscila Souza, Nikée Groot, Eric Arets, Victor Chama Moscoso, Wendeson Castro, Euridice N. Honorio Coronado, Marielos Peña-Claros, Clement Stahl, Jorcely Barroso, Joey Talbot, Ima Célia Guimarães Vieira, Geertje van der Heijden, Raquel Thomas, Vincent A. Vos, Everton C. Almeida, Esteban Álvarez Davila, Luiz E.O.C. Aragão, Terry L. Erwin, Paulo S. Morandi, Edmar Almeida de Oliveira, Marco B.X. Valadão, Roderick J. Zagt, Peter van der Hout, Patricia Alvarez Loayza, John J. Pipoly, Ophelia Wang, Miguel Alexiades, Carlos E. Cerón, Isau Huamantupa-Chuquimaco, Anthony Di Fiore, Julie Peacock, Nadir C. Pallqui Camacho, Ricardo K. Umetsu, Plínio Barbosa de Camargo, Robyn J. Burnham, Rafael Herrera, Carlos A. Quesada, Juliana Stropp, Simone A. Vieira, Marc Steininger, Carlos Reynel Rodríguez, Zorayda Restrepo, Adriane Esquivel Muelbert, Simon L. Lewis, Georgia C. Pickavance, Oliver L. Phillips

**Affiliations:** 1 School of Geography, University of Leeds, Leeds LS2 9JT, UK; 2 Jardín Botánico de Missouri, Prolongacion Bolognesi Mz.e, Lote 6, Oxapampa, Pasco, Peru; 3 Universidad Nacional de San Antonio Abad del Cusco, Av. de la Cultura No 733, Exeter Cusco, 733, Peru; 4 Geography, College of Life and Environmental Sciences, University of Exeter, Exeter EX4 4RJ, UK; 5 Environmental Change Institute, School of Geography and the Environment, University of Oxford, Oxford OX1 3QY, UK; 6 Naturalis Biodiversity Centre, PO Box 9517, 2300 RA Leiden, The Netherlands; 7 Ecology and Biodiversity, Institute for Environmental Biology, Utrecht University, Utrecht 80125, 3508 TC, The Netherlands; 8 Science and Education, The Field Museum, 1400 South Lake Shore Drive, Chicago, Illinois 60605–2496, USA; 9 Center for Tropical Conservation, Nicholas School of the Environment, Duke University, Box 90381, Durham, North Carolina 27708, USA; 10 INRA, UMR ‘Ecologie des Forêts de Guyane', Kourou Cedex 97387, France; 11 International Center for Tropical Botany, Department of Biological Sciences, Florida International University, Miami FL 33199, USA; 12 CNRS, UMR Ecologie des Forêts de Guyane, Kourou Cedex 97387, France; 13 CIRAD, UMR Ecologie des Forêts de Guyane, Kourou Cedex 97387, France; 14 Instituto Nacional de Pesquisas da Amazônia, Projeto Dinâmica Biológica de Fragmentos Florestais, Manaus, CEP 69080-971 AM, Brazil; 15 Centre for Tropical Environmental and Sustainability Science (TESS) and College of Marine and Environmental Sciences, James Cook University, Cairns, Queensland 4878, Australia; 16 Université Paul Sabatier CNRS, UMR 5174 Evolution et Diversité Biologique, Bâtiment 4R1, Toulouse 31062, France; 17 UAG, UMR Ecologie des Forêts de Guyane, Kourou Cedex 97387, France; 18 Department of Biology, University of Turku, Turku FI-20014, Finland; 19 Museu Universitário, Universidade Federal do Acre, Rio Branco 69910-900, Brazil; 20 UNELLEZ-Guanare, Programa de Ciencias del Agro y el Mar, Herbario Universitario (PORT), Mesa de Cavacas, Estado Portuguesa 3350, Venezuela; 21 Museo de Historia Natural Noel Kempff Mercado, Universidad Autonoma Gabriel Rene Moreno, Casilla 2489, Av. Irala 565, Santa Cruz, Bolivia; 22 INRA, UMR EEF, Champenoux 54280, France; 23 Universidad de Los Andes, Facultad de Ciencias Forestales y Ambientales, Mérida, Venezuela; 24 Universidad Estatal Amazónica, Facultad de Ingeniería Ambiental, Paso lateral km 2 1/2 via Napo, Puyo, Ecuador; 25 Universidade do Estado de Mato Grosso, Campus de Nova Xavantina, Caixa Postal 08, CEP 78.690-000, Nova Xavantina, MT, Brazil; 26 Museu Paraense Emilio Goeldi, C.P. 399, CEP 66040-170, Belém, Brazil; 27 Northeast Region Inventory and Monitoring Program, National Park Service, 120 Chatham Lane, Fredericksburg, Virginia 22405, USA; 28 Instituto Boliviano de Investigación Forestal, CP 6204, Santa Cruz de la Sierra, Bolivia; 29 Facultad de Ciencias Agrícolas, Universidad Autónoma Gabriel René Moreno, Santa Cruz de la Sierra, Bolivia; 30 Instituto de Ciencias Naturales, Universidad Nacional de Colombia, Apartado 7945, Bogotá, Colombia; 31 Alterra, Wageningen University, and Research Centre, PO Box 47, Wageningen 6700 AA, The Netherlands; 32 Van Hall Larenstein University of Applied Sciences, Velp, PO Box 9001, 6880 GB, The Netherlands; 33 World Wildlife Fund, 1250 24th Street NW, Washington, DC 20037, USA; 34 Forest Ecology and Forest Management Group, Wageningen University, PO Box 47, Wageningen 6700 AA, The Netherlands; 35 Tropenbos International, PO Box 232, Wageningen 6700 AE, The Netherlands; 36 Instituto Nacional de Pesquisas da Amazônia, Manaus, AM, CEP 69080-971, Brazil; 37 Programa de Pós-Graduação Ecologia e Manejo de Recursos Naturais, Universidade Federal do Acre, Rio Branco AC 69910-900, Brazil; 38 Instituto de Investigaciones de la Amazonia Peruana, Apartado 784, Iquitos, Peru; 39 INRA, UR 874, Research Unit on permanent grasslands, Clermont Ferrand 63100, France; 40 Universidade Federal do Acre, Campus de Cruzeiro do Sul, CEP 69920-900, Rio Branco, Brazil; 41 University of Wisconsin-Milwaukee, Milwaukee, Wisconsin 53211, USA; 42 Smithsonian Tropical Research Institute, Apartado Postal 0843-03092, Panama; 43 Iwokrama International Centre for Rainforest Conservation and Development, 77 High Street Kingston, Georgetown, Guyana; 44 Universidad Autónama del Beni, Campus Universitario, Av. Ejército Nacional, Riberalta, Bolivia; 45 Centro de Investigación y Promoción del Campesinado - Norte Amazanico, C/Nicanor Gonzalo Salvatierra Nu 362, Casilla 16, Riberalta, Bolivia; 46 Instituto de Biodiversidade e Floresta, Universidade Federal do Oeste do Pará, Santarém, PA CEP: 68.035-110, Brazil; 47 Servicios Ecosistémicos y Cambio Climático, Jardín Botánico de Medellín, Calle 73 N 51D - 14, Medellín, Colombia; 48 National Institute for Space Research, Avenida dos Astronautas, 1.758—Jd. Granja, São José dos Campos, SP CEP 12227-010, Brazil; 49 Smithsonian Institution, PO Box 37012, MRC 187, Washington, District of Columbia 20013-7012, USA; 50 Van der Hout Forestry Consulting, Jan Trooststraat 6, Rotterdam 3078 HP, The Netherlands; 51 UF-IFAS/Broward Co Extension Education, 3900 SW 100th Avenue, Davie, Florida, USA; 52 School of Earth Sciences and Environmental Sustainability, Northern Arizona University, Flagstaff AZ 86011, USA; 53 School of Anthropology and Conservation, Marlowe Building, University of Kent, Canterbury CT1 3EH, UK; 54 Herbario Alfredo Paredes (QAP), Universidad Central del Ecuador, Ciudadela Universitaria, Av. América, Quito, Ecuador, Quito, Ecuador; 55 Department of Anthropology, University of Texas at Austin, Austin, Texas 78712, USA; 56 Centro de Energia Nuclear na Agricultura, Universidade de São Paulo, São Paulo, CEP 05508-070, SP, Brazil; 57 Department of Ecology and Evolutionary Biology, University of Michigan, MI 48109, Ann Arbor, USA; 58 Centro de Ecologia, Instituto Venezolano de Investigaciones Cientificas, Carretera Panamericana, Km 11, Altos de Pipe, IVIC, Caracas, Venezuela; 59 ReforeST Group, DIHMA, Universidad Politécnica de Valencia, Valencia 46022 Spain; 60 European Commission—DG Joint Research Centre, Institute for Environment and Sustainability, Via Enrico Fermi 274, Ispra 21010, Italy; 61 Núcleo de Estudos e Pesquisas Ambientais, Universidade Estadual de Campinas, Campinas, SP CEP 13083-970, Brazil; 62 Conservation International, 2011 Crystal Drive, Suite 500, Arlington, Virginia 22202, USA; 63 Facultad de Ciencias Forestales, Universidad Nacional Agraria La Molina, Lima, Peru; 64 Department of Geography, University College London, London WC1E 6BT, UK

## Abstract

While Amazonian forests are extraordinarily diverse, the abundance of trees is skewed strongly towards relatively few ‘hyperdominant' species. In addition to their diversity, Amazonian trees are a key component of the global carbon cycle, assimilating and storing more carbon than any other ecosystem on Earth. Here we ask, using a unique data set of 530 forest plots, if the functions of storing and producing woody carbon are concentrated in a small number of tree species, whether the most abundant species also dominate carbon cycling, and whether dominant species are characterized by specific functional traits. We find that dominance of forest function is even more concentrated in a few species than is dominance of tree abundance, with only ≈1% of Amazon tree species responsible for 50% of carbon storage and productivity. Although those species that contribute most to biomass and productivity are often abundant, species maximum size is also influential, while the identity and ranking of dominant species varies by function and by region.

## Abstract (web summary)

The Amazon rainforest is dominated by relatively few tree species, yet the degree to which this hyperdominance influences carbon cycling remains unknown. Here, the authors analyse 530 forest plots and show that ∼1% of species are responsible for 50% of the aboveground carbon storage and productivity.

## Graphical abstract

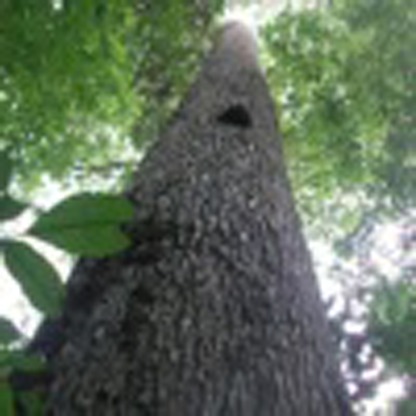

Amazonia still represents the largest tropical forest in the world, covering 5.3 million km^2^ (ref. 1), and accounting for 14% of carbon fixed by photosynthesis in the terrestrial biosphere^2^ and 17% of the terrestrial vegetation carbon stock^3^,^4^. Amazon forests also harbour the greatest diversity on the planet, with an estimated 16,000 tree species^1^. In spite of this great diversity, a relatively small minority of tree species are extremely common, with half of all the Amazonian trees accounted for by only 227 ‘hyperdominant' species, 1.4% of the estimated total^1^. Given the great concentration of diversity, carbon and metabolic activity in Amazonia, it is important we understand whether and how the phenomenon of hyperdominance may also influence the Amazon's carbon storage and cycling functions. For example, if Amazonia's substantial biomass carbon stocks (∼100 Pg C in aboveground live trees^4^) and biomass production are highly concentrated in few species, they may be less resilient to environmental change than would be expected given that high species diversity typically confers high resilience^5^. Likewise, improved understanding of how forest carbon stocks and cycling are linked to tree identity should lead to better informed predictions of forest carbon under future land-use and climate change scenarios.

It might be reasonably expected that exceptionally abundant taxa will dominate ecosystem function and hence strongly influence carbon cycling in Amazonia. However, the contribution each species makes to biomass stocks and wood production depends not only on its abundance, but also on the functional properties of the individual trees of the species. In particular, the size of a tree, its lifespan, growth rate and the density of its wood all determine how much carbon it stores and for how long. As the traits of individual trees are at least partially conserved at the species level (with additional variation determined by the local environment)^6^,^7^, the relative functional contributions of species may substantially vary from one species to another, independent of their abundance. Thus, some particularly abundant species may not in fact contribute substantially to biomass dynamics, whereas other much rarer taxa may do so.

The aim of this paper is to explore the concept of hyperdominance with respect to carbon cycling in Amazonian trees. Specifically, we use a large data set (Fig. 1), mostly from the RAINFOR network, to answer three questions: (i) are aboveground woody biomass (hereafter biomass) and aboveground woody productivity (hereafter productivity) disproportionately driven by a few taxa?; (ii) is the contribution of each species to biomass and productivity equal to its contribution to stem abundance? and (iii) to what extent do two species-level traits closely related to tree mass (maximum size and wood density) determine which species dominate stem abundance, biomass and productivity?

We find that (i) biomass and productivity are even more concentrated into few species than is stem abundance; (ii) species contributions to biomass and productivity are significantly related, but not equal to, contributions to stem abundance and (iii) large species contribute disproportionately more to biomass and productivity.

## Results

### Number of hyperdominant species

Just 182 species, or 5.3% of the 3,458 identified species in the data set, were classed as biomass hyperdominants (that is, those species that collectively account for 50% of biomass). Only 184 species, or 6.4% of the 2,883 identified species in the productivity data set, were classed as productivity hyperdominants (Table 1). Rather more species, 283 or 8.2%, were required to account for 50% of stem numbers. The top 20 highest biomass species are given in Table 2, and the top 20 species by stem abundance and productivity are listed in Supplementary Tables 1 and 2. The abundance, biomass and productivity of all species in the data set are provided as a data package ( DOI: 10.5521/FORESTPLOTS.NET/2015_1).

### Characteristics of hyperdominant species

The stem hyperdominant species contribute considerably to the total biomass and productivity, albeit with considerable scatter (Fig. 2). The relative contribution of a species to the total number of stems was a good predictor of its contribution to total biomass (F=12,360, df=3,456, *P*<0.0001, *R*^2^=0.78 (F—F-test statistic for predictor significance, df—degrees of freedom, *P*—probability of result occurring by chance, *R*^2^—coefficient of determination)) and productivity (F=5,425, df=2,804, *P*<0.0001, *R*^2^=0.66) with all variables on a log scale. Yet, among hyperdominants, the individual ranking of importance in terms of stem abundance is a poor predictor of its functional contribution—of the top 20 stem hyperdominants, most are absent from the equivalent top biomass and productivity lists (Table 2 and Supplementary Tables 1 and 2). Species contributions to abundance were effectively independent both of maximum *D* and of wood density because, although significant relationships were found, the *R*^2^ was very low (0.07 and 0.03 for maximum *D* and wood density respectively, Supplementary Fig. 1). This inference is further supported by the close match between curves of cumulative % contribution to stem abundance and cumulative % of species from high to low trait values (Fig. 3), and by the observation that the species with highest 50% of wood density and the largest 50% of species each contribute close to 50% of stems (Table 3).

Independent of the abundance effect, species contributions to biomass and productivity were also strongly related to their maximum *D* (Fig. 4). Thus, large species contributed disproportionately both to biomass and to productivity, with the largest 50% of species contributing 82.5% and 79.8% of biomass and productivity, respectively (Table 3 and Fig. 3a). As a result, the cumulative % contribution curves from high to low maximum *D* for biomass and productivity were shifted to the left compared with the species and stem curves (Fig. 3a). In addition, after stem abundance was accounted for, maximum *D* was a highly significant predictor of species contributions to biomass (F=6,218, df=1,317, *P*<0.0001, *R*^2^=0.83, Fig. 4a) and productivity (F=2,577, df=1,254, *P*<0.0001, *R*^2^=0.67, Fig. 4b). However, after accounting for stem abundance, wood density had no relationship with species contributions to productivity (F=1.8, df=1,186, *P*=0.18, *R*^2^=0.0006, Fig. 4d), with a weak relationship found with species contributions to biomass (F=74.77, df=1,301, *P*<0.0001, *R*^2^=0.054, Fig. 4c). The somewhat higher contribution to biomass by species with dense wood is shown by the leftward shift in the cumulative % curve in Fig. 3b, whereas the curve for productivity roughly follows those of species and stems. The 50% of species with the densest wood make up 64.7% of biomass, but only 53.6% of productivity.

### Regional patterns

Species classed as hyperdominants across the whole data set were typically hyperdominant in just one or two of the five regions (Fig. 5). This geographic patterning was strongest for biomass and productivity hyperdominants, for which 82.4% and 88.0% of species were dominant in only one or two regions, compared with 70.7% for stem hyperdominants. 12.4% of stem hyperdominants were not classed as hyperdominants in any region, compared with 4.9% and 1.1% of biomass and productivity hyperdominants, respectively. Within regions, typically a higher percentage of species were classed as hyperdominants in all categories (Table 1), compared with the Amazon-wide analysis. The relationships between stem contributions and biomass and productivity contributions followed similar patterns to the Amazon-wide analysis, as did the patterns with maximum *D* and wood density (Figs 6 and 7, Supplementary Figs 2–7 and Supplementary Table 3). However, the explanatory power of the statistics was typically lower for the analyses based on regional data sets, with lower *R*^2^ values for the regressions (Figs 6 and 7, Supplementary Figs 5–7 and Supplementary Table 3). In general, the analyses had more explanatory power in the Guiana Shield, East-Central and Southwestern regions than the Brazilian Shield and Northwestern regions.

## Discussion

We find that ‘hyperdominance' (the phenomenon of disproportionate influence of a small fraction of species) is remarkably strong for the vital forest functions of carbon storage and woody productivity, with 182 biomass and 184 productivity hyperdominant species, compared with 283 for stem abundance (Table 1). As expected, abundant species do contribute greatly to forest biomass stocks and productivity, with 78% of variation in species contributions to biomass and 66% of variation in species contributions to productivity explained by species' relative stem abundance (Fig. 2, all variables on a log scale). However, the contribution of a species to stem abundance differs substantially from its contribution to the measured ecosystem functions. For instance, only five species are top 20 contributors to each of stem abundance, biomass and productivity (Table 2, Supplementary Tables 1 and 2), and approximately one-third of the biomass and productivity hyperdominant species do not even register as stem hyperdominants, despite the stem hyperdominant list containing many more species. The clearest example of a mismatch between abundance and biomass contribution is the species *Dinizia excelsa* (Ducke). Despite being ranked in position 931 in terms of stem abundance (with just 31 stems), *D. excelsa* ranks 24th by biomass, contributing 0.45% of the total. The mismatch is due to the species' traits; extreme maximum size (165 cm *D*) and wood density (0.94 g cm^−3^) together explain why *D. excelsa* can contribute so much biomass with so few stems.

**Figure 1:**
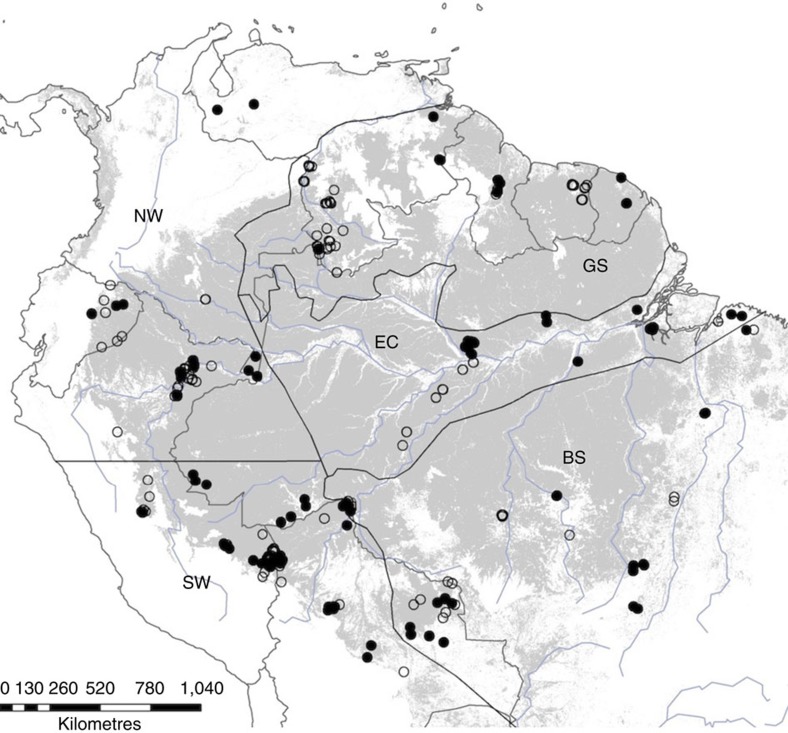
Map of plot locations. Open circles—single census plots used for biomass and stem number analyses, closed circles—multi-census plots used for biomass, productivity and stem number analyses. Black lines—Amazon regional boundaries from Feldpausch *et al.*^36^ with additional north–south separation of the western Amazon; BS—Brazilian shield, EC—east central, GS—Guiana shield, NW—north western, SW—south western. Grey—unflooded closed canopy forest below 500 m.a.s.l. reclassified from GLC2000 data^41^.

**Figure 2:**
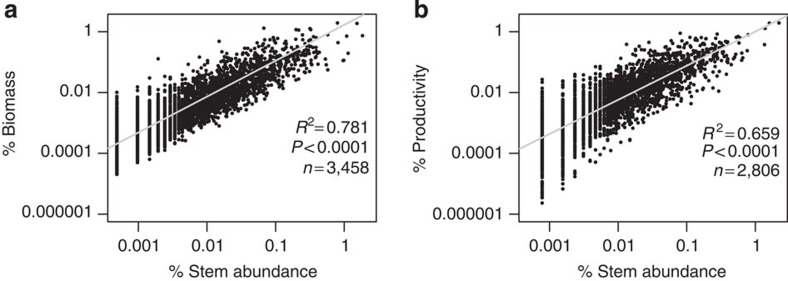
Relationships between species contributions to stem abundance and contributions to biomass and productivity. % contribution of species to total stem abundance with % contribution to (**a**) total aboveground biomass and (**b**) total aboveground woody productivity. Regression models are plotted with grey lines. Regression equation for % contribution to biomass: log(% biomass)=0.22+1.18 log(% stem), regression equation for productivity: log(% productivity)=0.003+1.12 log(% stem). All 530 plots are used for **a**, and the reduced productivity data set of 221 plots is used for **b**. 77 species with negative or 0 productivity were excluded from **b**. Plotted on log scale.

We find 283 stem hyperdominant species in the RAINFOR data set, more than the 227 found by ter Steege *et al.*^1^ Two likely reasons for this are, first, that our analysis concentrates on well-drained forests typical of Amazonia, whereas the ter Steege *et al.* analysis also included the seasonally flooded and swamp forest types that are typically much less diverse^8^, and second, we did not attempt to account for the spatial distribution of our plots across Amazonia (Fig. 1). Hence, the precise lists of species cannot be taken as a robust estimate of the most dominant species in Amazonia, but rather the species that dominate within our data set. However, this does not affect the suitability of the data for our aims of assessing the relationship between species abundance and contribution to forest function, which is only possible with a widespread plot network with careful botanical identifications and monitored through time. Using the ter Steege *et al.*^1^ estimated number of stem hyperdominant species, and assuming that the ratio of stem/biomass and stem/productivity hyperdominants we find is representative of Amazonia, we can estimate that there would be 147 biomass and 167 productivity hyperdominant species across Amazonia. Considering the estimated 16,000 tree species in the Amazon^1^, this implies that half of the carbon stock and half of the woody productivity are controlled by just ≈1% of species, respectively.

We find that for all categories, hyperdominant species are most commonly only dominant in a single Amazon region (Fig. 5). However, stem hyperdominants were more evenly spread across regions than biomass and productivity hyperdominants. In particular, many more stem hyperdominant species (29.3%) than biomass (17.6%) and productivity (12.0%) hyperdominant species were dominant in three or more regions, or not dominant in any. The data therefore suggest that environmental conditions act as much stronger constraints on the ability of a species to dominate a community's metabolism than simply to persist in it.

**Figure 3:**
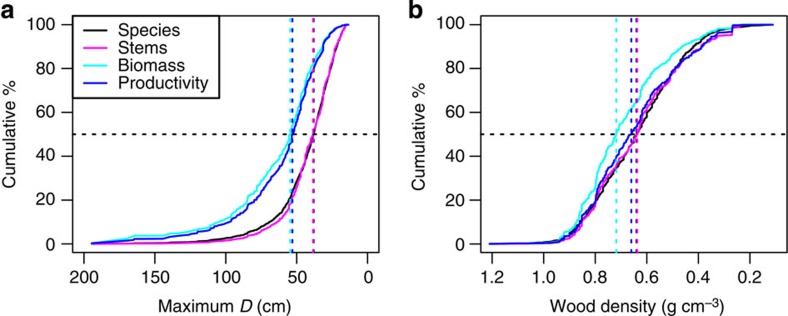
Cumulative % contribution to species, stems, biomass and productivity ordered by maximum *D* and wood density. (**a**) Maximum *D* (*n*=1,256), (**b**) wood density (*n*=1,188). Horizontal dashed black lines represent the mid-point of all metrics, vertical dashed lines show the trait value at the mid-point of each metric. All curves are based on the reduced productivity data set, curves for biomass and stems are very similar when using the full data set (data not shown).

**Figure 4:**
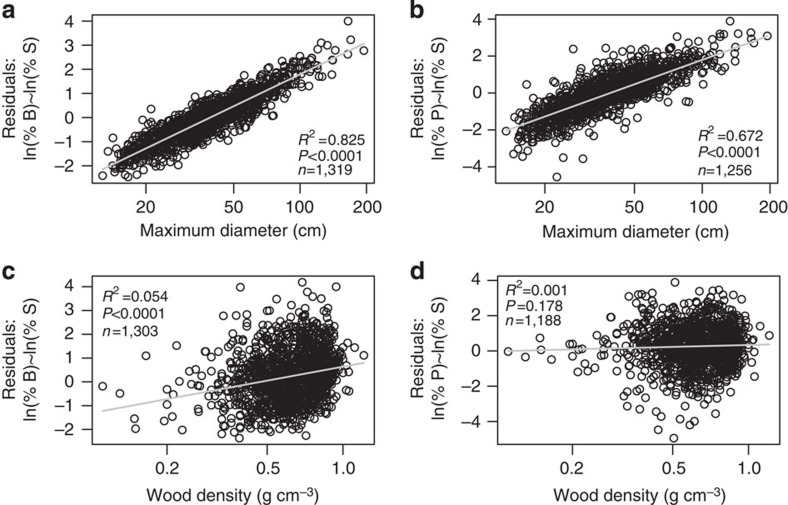
Patterns between plant traits and contributions to biomass and productivity after accounting for abundance. Relationship between the residuals from ln(% contribution to biomass)=*a*+*b* * ln(% contribution to stem number) and (**a**) maximum *D* and (**c**) wood density, relationships between the residuals from ln(% contribution to productivity)=*a*+*b* * ln(% contribution to stem number) and (**b**) maximum *D*, and (**d**) wood density. Regression models are plotted with grey lines. Maximum diameter and wood density plotted on a log scale.

**Figure 5:**
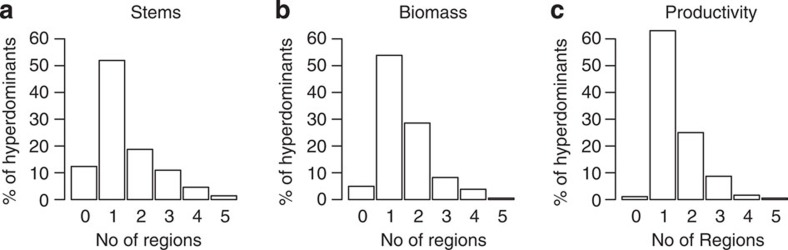
Percentage of Amazon-wide hyperdominant species that are also dominant within regions. (**a**) stem hyperdominants (*n*=283), (**b**) biomass hyperdominants (*n*=182), (**c**) productivity hyperdominants (*n*=184).

**Figure 6:**
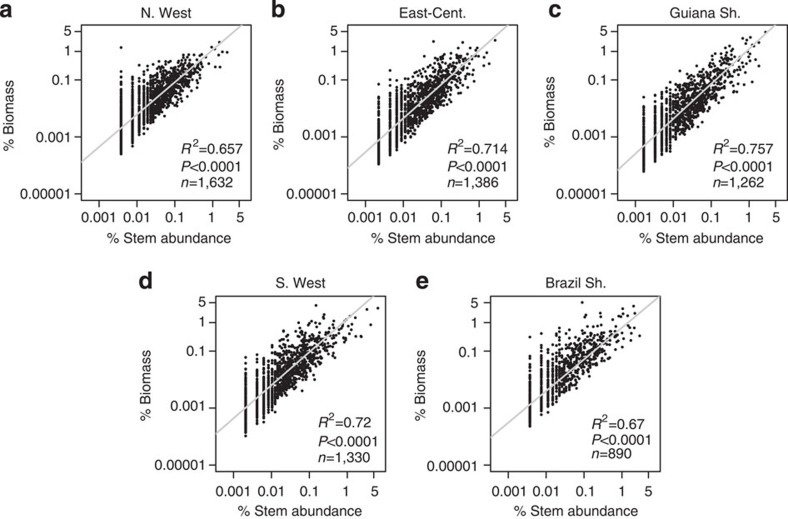
Relationships between % contribution of species to stems and % contribution to biomass in five different Amazon regions. (**a**) Northwestern Amazonia (N.West), (**b**) East-central Amazonia (East-Cent.), (**c**) Guiana shield (Guiana Sh.), (**d**) Southwestern Amazonia (S.West), (**e**) Brazilian shield (Brazil Sh.). Regression models are plotted with grey lines. Plotted on log scale.

**Figure 7:**
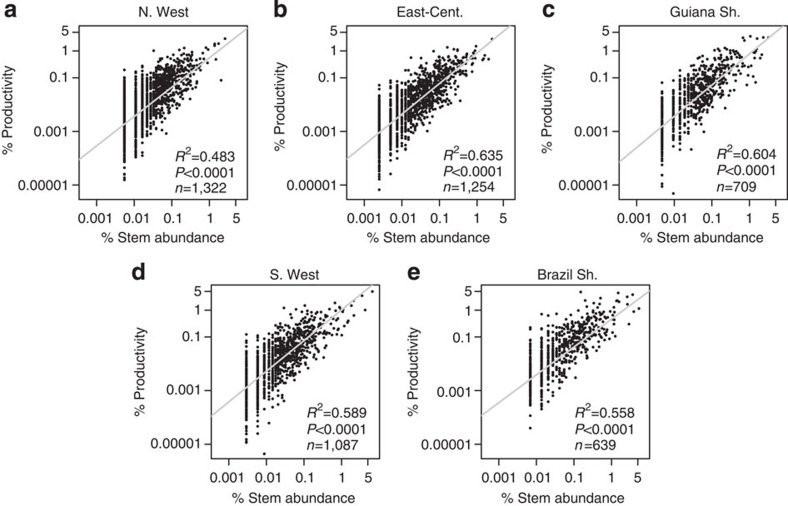
Relationships between % contribution of species to stems and % contribution to productivity in five different Amazon regions. (**a**) Northwestern Amazonia (N.West), (**b**) East-central Amazonia (East-Cent.), (**c**) Guiana shield (Guiana Sh.), (**d**) Southwestern Amazonia (S.West), (**e**) Brazilian shield (Brazil Sh.). Regression models are plotted with grey lines. Plotted on log scale.

Given the significance of the Amazon forest for the global carbon cycle, an understanding of the nature of dominant species and their potential sensitivity to future climate and anthropogenic disturbance is needed. We find that, after stem abundance of species is accounted for, maximum size is an excellent predictor of species contribution to biomass and productivity (Fig. 4), whereas maximum size was not a good predictor of species relative abundances (Fig. 3 and Supplementary Fig. 1). One might expect that small-sized species would be disproportionately abundant compared with large species (for example, a negative slope in Supplementary Fig. 1) because forests are composed overwhelmingly of small stems. However, our results show this is not so. Species with small maximum size do not contribute disproportionately to total stem abundance; rather, many species are small and most Amazonian tree diversity is focused in understory and sub-canopy taxa. In contrast, the species with the potential to grow to large sizes contribute disproportionately to biomass and productivity, with the greatest skew in the case of biomass. Large volume trees tend to have greater mass, and their height and greater leaf area also allow greater access to light and the potential for high rates of carbon fixation and biomass growth^9^.

We find little evidence to support wood density being an important correlate of abundance among Amazon species (Fig. 3 and Supplementary Fig. 1), consistent with the findings of ter Steege *et al.*^1^ Although the relationship between wood density and the contribution to stem abundance was marginally significant, it had very low explanatory power. Similarly, there was a marginally significant but weak association between species contribution to biomass and wood density, and no relationship with contribution to productivity (Figs 3 and 4). The lack of a relationship with productivity is consistent with observations at the individual level^10^.

Just two variables, species relative abundance and species maximum size, account for 96% of the variation in species contributions to the total biomass stock in the data set (with all variables on a log scale). Although the variation explained by these two variables for species contributions to productivity was also very high (87%), additional plant traits such as those related to resource acquisition and the leaf economics spectrum^11^, for example, maximum photosynthetic rate, presumably also play some role. When analysed on a regional basis, abundance and maximum size were better predictors of species contributions to biomass and productivity in the Guiana Shield, East-Central and Southwestern Amazon regions than the Brazilian Shield and Northwestern regions (Supplementary Figs 6 and 7). This may be due to lower sample sizes in the Brazilian Shield and Northwestern regions, or due to real differences in forest physiology.

Although the significance of individual large trees for forest biomass is not necessarily surprising and has been documented before^12^,^13^,^14^, we here establish this relation at the species level, across the Amazon terra firme forests and, crucially, extend it to productivity. Large trees also perform other important ecological roles in forests, yet face a myriad of threats^15^ such as harvesting, forest fragmentation^16^ and climate change^17^,^18^,^19^,^20^. With one-third of the forest biomass stock stored by the largest 10% of species, understanding the sensitivity to environmental change of these taxa is clearly important. Moreover, the concentration of function into a relatively small number of taxa does potentially help simplify attempts at modelling the current ecophysiology of Amazon forests. Data on the functional traits of key hyperdominant species could be used to inform next-generation trait-based dynamic vegetation models^21^,^22^. However, there are clearly complications due to regional differences between dominating species.

More broadly, although a small fraction of Amazon tree species contribute disproportionately to carbon storage and cycling, and remarkably so, this does not necessarily indicate that high diversity levels are immaterial for ecosystem function. For instance, our analysis represents a snap-shot of recent Amazon diversity and function for trees which mostly became established under twentieth century climates, whereas under future conditions a different suite of species may dominate. Rare species are thought to possess uncommon combinations of functional traits^23^ and therefore may be important for the full spectrum of responses to altered conditions. Tropical forest species composition is known to be dynamic and potentially responsive to environmental changes^24^,^25^,^26^,^27^, but for this to be possible the future dominant species, which may flourish under new conditions, must be present in the species pool. Thus, the very strong concentration of function into relatively few taxa today does not mean that high species-richness is irrelevant for the long-term survival and health of tropical forests, as biodiversity may act as an insurance against environmental variation.

In summary, we find that carbon in the world's most extensive and diverse tropical forest is concentrated into remarkably few species. Although the most abundant species contribute significantly to this phenomenon, other properties also govern which taxa are important for biomass dynamics. Notably, the maximum potential size of Amazon tree species is a key predictor of their capacity to store and gain carbon. Functional hyperdominance also has a strong geographical signal. Thus, most species that contribute strongly to carbon cycling only do so within one region within Amazonia.

## Methods

### Data sets

We used a data set of 530 sample plots located in the Amazon region (Fig. 1) compiled in the RAINFOR data set^28^,^29^ and curated at ForestPlots.net^30^. This data set includes a number of plot networks including Tropical Ecology Assessment and Monitoring, PPBio (Brazilian Program for Biodiversity Research) and the Alwyn H. Gentry Forest Transect Dataset. Many of the plots are also included in the Amazon Tree Diversity Network used by ter Steege *et al.*^1^ We restricted the analysis to sites below 500 m.a.s.l., in old growth forests (excluding any successional, burnt or logged), occurring on terra firme substrate (excluding swamp and seasonally flooded forests) and excluding cerrado. This allowed us to minimize the possible influence of rare species restricted to rarer and poorly sampled forest types and to ensure that we restricted our questions to the dominant Amazon formations growing on unflooded terrain. The data set consists of repeated measurements of tree diameter (*D*; diameter at 1.3 m or above buttresses) and species identity of all trees ≥10 cm *D*, following a standard protocol^31^. The mean plot size was 0.69 ha (range 0.04–25.0 ha). All recorded species names were checked against the Tropicos database using the Taxonomic Name Resolution Service (TNRS v3.2 (ref. 32)) and corrected as necessary. Morphospecies were considered to be unidentified. Wood density values were taken from the Global Wood Density Database^33^,^34^. The 530 plot data set contained 206,135 trees from 3,458 species, consisting of 114,696 Mg of biomass. For productivity analysis, we used a subset of 221 multiple census plots with at least 2 years between the initial and final censuses, in total accounting for 1,231 Mg biomass per year of aboveground woody productivity. Finally, all analyses were repeated on a data set restricted to 326 plots (148 plots for productivity), where at least 80% of stems within the plot were identified to species, in order to test whether the level of identification in the data set influenced results (see Supplementary Figs 8–11 and Supplementary Tables 4–8 for results based on this data set).

### Data analysis

We treated our data as a sample of the terra firme forests of Amazonia and analysed the data set as a whole, rather than at the plot level. Stem abundance and biomass of each species were calculated using the first census of each plot (across all plots 79.0% of all stems were identified to species). Species-level stem abundance was calculated as the total number of stems of a species. Species-level biomass was calculated as the sum of biomass of all stems of a species. Stem-level biomass was calculated using the moist forest biomass equation based on diameter, wood density and height from Chave *et al.*^35^, with height based on the region-specific Weibull equations from Feldpausch *et al.*^36^ For monocots (families Arecaceae and Strelitziaceae), an Arecaceae-specific equation was used to estimate biomass from diameter only^37^.

For productivity, we used the 221 multi-census plot data set (‘productivity data set') and only the stems alive in the first census of each plot (for consistency with the stem abundance and biomass analyses). Mean stem-level productivity (*P*_stem_) was calculated as the mean annual productivity of each stem across all census intervals for which it was present.


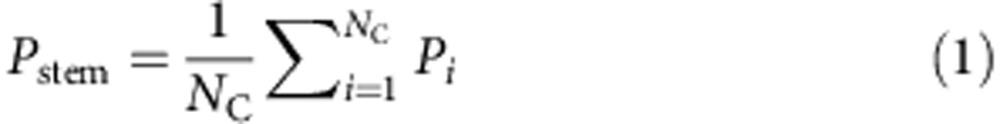


where *N*_C_ is the number of censuses for which an individual stem is alive for, *P*_*i*_ is the productivity of a stem in census interval *i*. We include the productivity of stems in the census interval in which they recruited, assuming a *D* of 10 cm at the beginning of the census interval. In cases where the point of measurement (POM) was changed between censuses, we used the diameter at a standardized POM to avoid artefacts associated with disjoint diameter sequences^38^. To estimate productivity of a species across all plots (*P*_species_), we summed the productivity of each stem of that species. See Talbot *et al.*^39^ for a discussion of the estimation of productivity; the methods used here are the equivalent of *R*_2_ (for recruits) and *G*_2_ (for POM changes) in Talbot *et al.*^39^ In cases where individuals subsequently died in the second plot census, it was not possible to estimate productivity for these stems. In some cases, this was true of all stems of a species (2.2% of species). Hence, the species contributions to productivity are based on a slightly smaller number of trees than contributions to stem abundance and biomass. We assume that the mortality is evenly spread between species and therefore that species relative contributions to total stems, biomass and productivity should not be affected.

For monocot stems, which lack radial growth, we used an alternative method to estimate productivity as repeated height measurements were not available. Biomass for palms can be reasonably estimated using diameter measurements, with few species-specific biases^37^. Therefore, we used an alternative method by estimating necromass production. This method requires an adequate sample of stems so we limited the analysis to the monocot species classed as stem hyperdominants and hence productivity of rare palms was not estimated. We assumed that the populations of each palm species are in approximate equilibrium, such that the long-term stem biomass mortality rates equal long-term stem biomass production rates. We derived the stem necromass production rates for each palm tree that died, based on its standing biomass (using the allometric model from Goodman *et al.*^37^) estimated from its last recorded *D*, allocated equally over the time period from the initial plot census date to the census date in which it died. As the dicot productivity estimates do not include the 10-cm *D* inner cylinder of the stem, for equivalence the biomass before death used in the calculation was reduced by the biomass estimate of a 10-cm *D* palm. Hence,


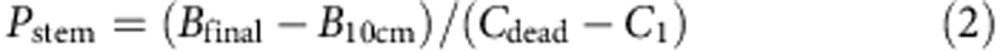


where *B*_final_ is the biomass estimated using last *D* measured for the stem, *B*_10cm_ is the biomass of a 10-cm palm, *C*_1_ is the initial census date and *C*_dead_ is the census date in which the palm was recorded as dead. Palm species productivity was then calculated as the sum of *P*_stem_ across all dead trees of the species.

Trees not identified to species level were used only to determine the denominator for the relative contribution of each identified species to the total data set. Species-level stem abundance and biomass relative contributions were calculated twice, once using the full 530 plot data set and once using the 221 plot productivity data set for use in further analyses comparing between measures.

To address the first question ‘are biomass and productivity also dominated by few taxa?', we determined the minimum number of species required to account for 50% of total stems, biomass or productivity in our plots. For simplicity, we term the species contributing 50% of stems ‘stem hyperdominants', the species contributing 50% of biomass ‘biomass hyperdominants' and the species contributing 50% of productivity ‘productivity hyperdominants'.

To address the second question ‘is the contribution of each species to biomass and productivity equal to its contribution to stem abundance?', we calculated the contribution of the stem hyperdominants to the total biomass and productivity of the data set. For biomass, this was based on the full data set, whereas for productivity this was based on the productivity data set, with stem hyperdominant species also defined using the productivity data set to ensure consistency between the species measures. Further, we regressed the percentage contribution of each species to biomass and productivity against their percentage contribution to stems. The regressions were performed using the full data set for biomass, and the productivity data set for productivity. Data were not normally distributed and therefore were log-transformed before analysis.

To address the third question ‘to what extent do maximum diameter and wood density determine which species dominate stem abundance, biomass and productivity?', we first calculated maximum *D* as the 95th percentile value for each species with at least 20 individuals included in the full 530 plot data set (and from any census, in total 1,319 species). Only the maximum of all diameter measures of an individual stem was used in the estimation of species maximum *D*. We then ordered the data set from highest to lowest trait value (maximum *D* or wood density) and plotted the cumulative percentage of species, stems, biomass and productivity against the trait value, and determined the contribution of the largest and highest wood density species to the different measures. Only the 1,303 species for which a species-specific wood density was available were included in the wood density analysis. In addition, we regressed the residuals from the linear model predicting percentage contribution to biomass or to productivity from percentage contribution to stems (see above) against trait value to examine the relationships with trait values when abundance is accounted for. These analyses were performed on the full data set for biomass and the reduced data set for productivity. To test for a relationship between species contribution to stem abundance and trait values, we regressed trait values against percentage contribution to stem abundance. Maximum *D* and wood density values were only available for approximately one-third of species in the data set, with rare species typically being those without a value. Although this exclusion of many rare species in this analysis could introduce unknown biases to the results, it also excludes additional noise in the data set from including species that have not been adequately sampled.

### Regional analysis

To investigate if the patterns found within the whole data set were consistent within different Amazon regions and to find out how the hyperdominant species are spread between regions, we repeated all analyses at the regional level. We used the Feldpausch *et al.*^36^ region delimitation based on substrate maximum geological age that was also used for height allometry (Guiana Shield, Brazilian Shield, East-Central and Western Amazonia), but further split the Western Amazon region at −8° latitude into Northwestern Amazon and Southwestern Amazon, following a similar delimitation by ter Steege *et al.*^1^ that separates the mostly aseasonal north from the more seasonal south. Species required to reach 50% of a regions stems/biomass/productivity were considered regional hyperdominants.

### Unidentified stems

Stems in the data set that were not identified to species-level were treated slightly differently. In hyperdominance calculations, these stems were used only to determine the denominator (total stems, biomass and productivity in the data set) in the estimation of known species contributions. To estimate their biomass and productivity, a wood density value is required. Wood density values for such stems were applied at the genus- or family-level, if known. For stems with no family-level identification, or where no wood density value was available for the species, genus or family, we applied the plot mean wood density value. Unidentified stems were excluded from further analyses. Because we include unidentified stems in hyperdominance calculations, the percentage of species necessary to account for 50% of total stems/biomass/productivity will be a slightly over-estimated as the exact total number of species in the data set is unknown because of incomplete botanical identifications.

All analyses were carried out in R version 2.15.1 (ref. 40).

## Author contributions

O.L.P., Y.M. and Jon Lloyd conceived the RAINFOR forest census plot network programme, S.F. wrote the paper, O.L.P., T.R.B., M.G., Y.M., M.O.J and S.F. conceived and designed the study, S.F., M.O.J. and O.L.P. carried out the data analysis, O.L.P., R.J.W.B., T.R.F, T.R.B., A.M.M. and G.L.G. coordinated data collection with the help of most co-authors. All co-authors collected field data and commented on or approved the manuscript.

## Additional information

**Accession codes:** The permanently archived data package of the species-level data set ( DOI: 10.5521/FORESTPLOTS.NET/2015_1) can be accessed at https://www.forestplots.net/data-packages/fauset-et-al-2015.

**How to cite this article:** Fauset, S. *et al.* Hyperdominance in Amazonian forest carbon cycling. *Nat. Commun.* 6:6857 doi: 10.1038/ncomms7857 (2015).

## Supplementary Material

Supplementary Information Supplementary Figures 1-11 and Supplementary Tables 1-8

## Figures and Tables

**Table 1:** Hyperdominance of stem abundance and carbon cycling in the Amazon.

	**Full data set**				**Productivity data set**				
	**Plots**	**Species**	**No. of hyperdominants (%)**		**Plots**	**Species**	**No. of hyperdominants (%)**		
			**Stems**	**Biomass**			**Stems**	**Biomass**	Productivity*
Amazon-wide	530	3,458	283 (8.2)	182 (5.3)	221	2,965	250 (8.4)	160 (5.4)	184 (6.4)
Northwestern	123	1,632	199 (12.2)	170 (10.4)	33	1,412	162 (11.5)	138 (9.8)	115 (8.4)
Southwestern	169	1,330	60 (4.5)	64 (4.8)	59	1,185	62 (5.2)	62 (5.2)	66 (5.8)
Guiana Shield	116	1,262	131 (10.4)	62 (4.9)	49	748	92 (12.3)	36 (4.8)	52 (7.1)
East-Central	69	1,386	157 (11.3)	101 (7.3)	56	1,317	152 (11.5)	96 (7.3)	117 (9.1)
Brazilian Shield	53	890	82 (9.2)	55 (6.2)	26	698	39 (5.6)	23 (3.3)	30 (4.5)

Number and percentage of species that contribute 50% of stem numbers, aboveground biomass and aboveground productivity for the whole data set and split by region.

^*^If a tree dies before the second census, it will contribute to biomass and stems but will not have a productivity value, hence the percentage value is calculated from a slightly smaller total number of species (2,883).

**Table 2:** Top 20 most dominant species by aboveground woody biomass.

**Family**	**Species**	**Biomass (Mg)**	**% Total biomass**	**Cumulative % biomass**	**Rank by stem abundance**	Rank by productivity*
Fabaceae	*Eperua falcata*	2,217	1.93	1.93	8	8
Lecythidaceae	*Eschweilera coriacea*	2,142	1.87	3.80	2	2
Lecythidaceae	*Bertholletia excelsa*	1,498	1.31	5.11	243	4
Vochysiaceae	*Qualea rosea*	1,452	1.27	6.37	30	88
Lauraceae	*Chlorocardium rodiei*	1,340	1.17	7.54	71	13
Fabaceae	*Vouacapoua americana*	1,340	1.17	8.71	27	5
Goupiaceae	*Goupia glabra*	1,299	1.13	9.84	61	10
Burseraceae	*Tetragastris altissima*	908	0.79	10.64	10	6
Fabaceae	*Dicorynia guianensis*	898	0.78	11.42	56	16
Arecaceae	*Iriartea deltoidea*	847	0.74	12.16	1	1
Moraceae	*Pseudolmedia laevis*	819	0.71	12.87	4	3
Lecythidaceae	*Eschweilera sagotiana*	784	0.68	13.55	22	62
Sapotaceae	*Pradosia cochlearia*	736	0.64	14.19	176	275
Chrysobalanaceae	*Licania alba*	724	0.63	14.83	17	90
Caryocaraceae	*Caryocar glabrum*	689	0.60	15.43	149	50
Apocynaceae	*Aspidospermaexcelsum*	648	0.57	15.99	74	14
Sapotaceae	*Pouteria guianensis*	625	0.54	16.54	55	53
Fabaceae	*Swartzia polyphylla*	624	0.54	17.08	203	19
Fabaceae	*Dicymbe altsonii*	623	0.54	17.62	233	9
Olacaceae	*Minquartia guianensis*	623	0.54	18.17	29	21

^*^Productivity ranks are based on the 221 plot productivity data set.

**Table 3:** Contributions to total stems, biomass and productivity from largest and most densely wooded 50% of species.

	**% Contribution by largest 50% of species**	**Maximum** D* **at 50% of metric (cm)**	**% Contribution by 50% most densely wooded species**	**Wood density**† **at 50% of metric (g** **cm**^−3^)
Stems	50.5	38.5	49.7	0.64
Biomass	82.5	54.5	64.7	0.72
Productivity	79.8	53.0	53.6	0.66

^*^Median maximum diameter across all species: 38.0 cm.

^†^Median wood density across all species: 0.64 g cm^−3^.
