# Supplementary material for: Hyperdominance in Amazonian forest carbon cycling
Source: Nat Commun. 2015 Apr 28;6:6857. doi: 10.1038/ncomms7857 (PMC4423203; doi:10.1038/ncomms7857)
Supplement: Supplementary Information — Supplementary Figures 1-11 and Supplementary Tables 1-8 [file ncomms7857-s1.pdf]

Supplementary Figures

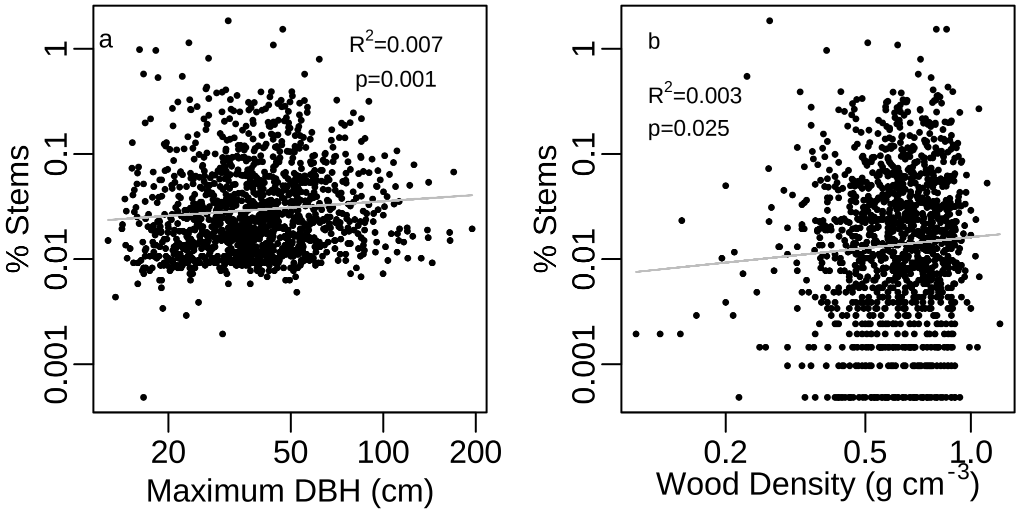

Supplementary Figure 1. Relationships between % contribution of species to stems and functional traits. (**a**) maximum *D* (n = 1319), (**b**) wood density (n = 1303). Regression models are plotted with grey lines. Plotted on log scale.

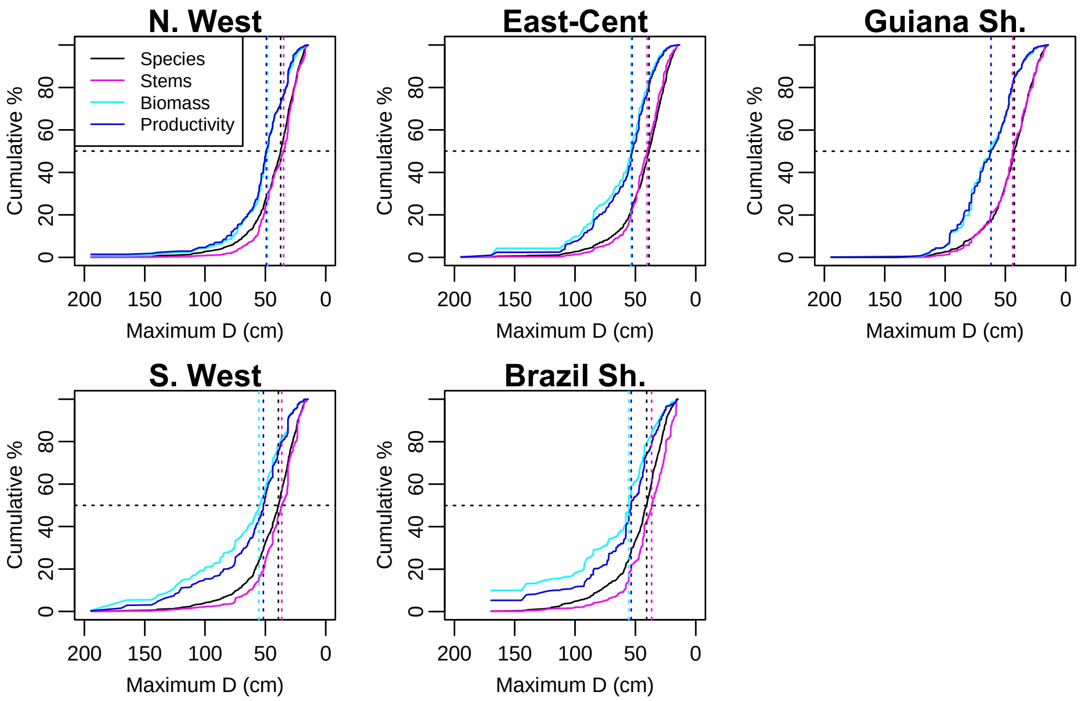

Supplementary Figure 2. Cumulative % contribution to species, stems, biomass and productivity ordered by maximum *D.* Horizontal dashed black lines represent the mid-point of all metrics, vertical dashed lines show the trait value at the mid-point of each metric. All curves are based on the productivity plot datasets.

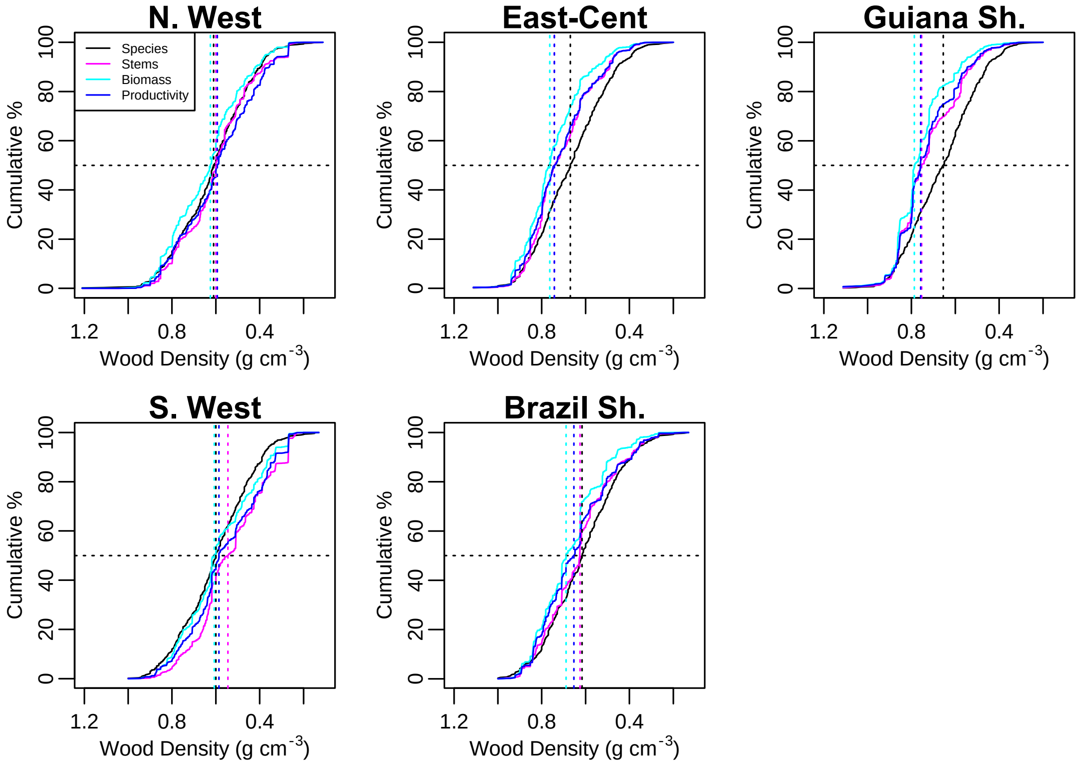

Supplementary Figure 3. Cumulative % contribution to species, stems, biomass and productivity ordered by wood density. (a) North western Amazonia, (b) East-central Amazonia, (c) Guiana shield, (d) South western Amazonia, (e) Brazilian shield. Horizontal dashed black lines represent the mid-point of all metrics, vertical dashed lines show the trait value at the mid-point of each metric. All curves are based on the productivity plot datasets.

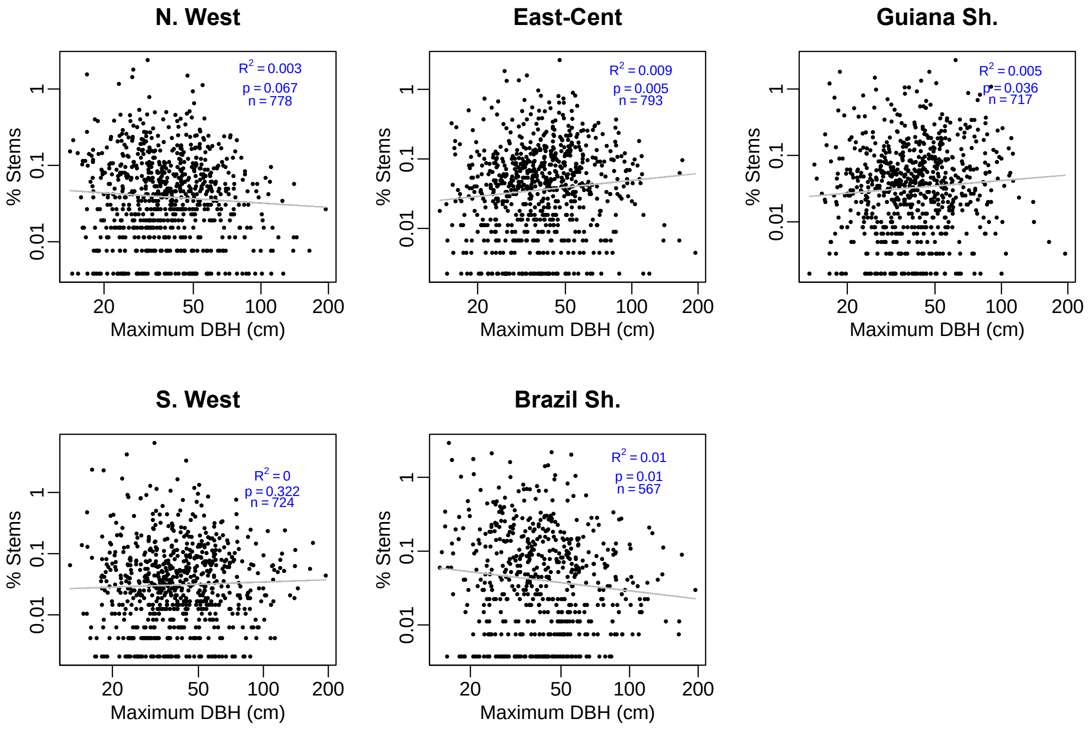

Supplementary Figure 4. Relationships between % contribution of species to stems and maximum D in five Amazon regions. Regression models are plotted with grey lines. Plotted on log scale.

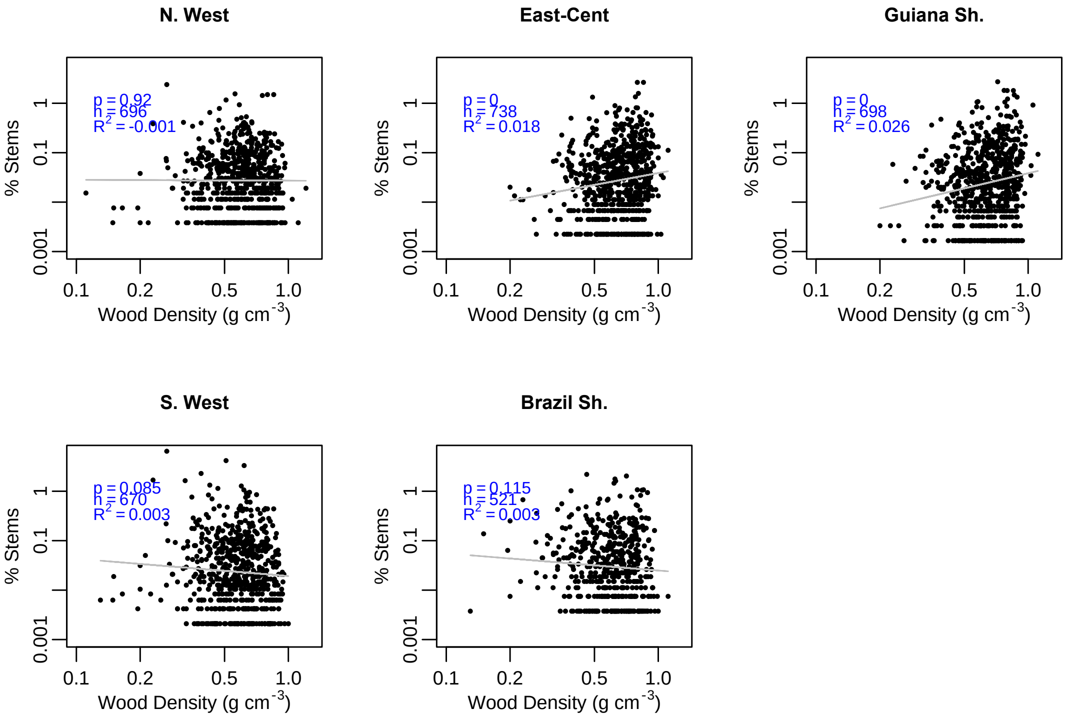

Supplementary Figure 5. Relationships between % contribution of species to stems and wood density in five Amazon regions. Regression models are plotted with grey lines. Plotted on log scale.

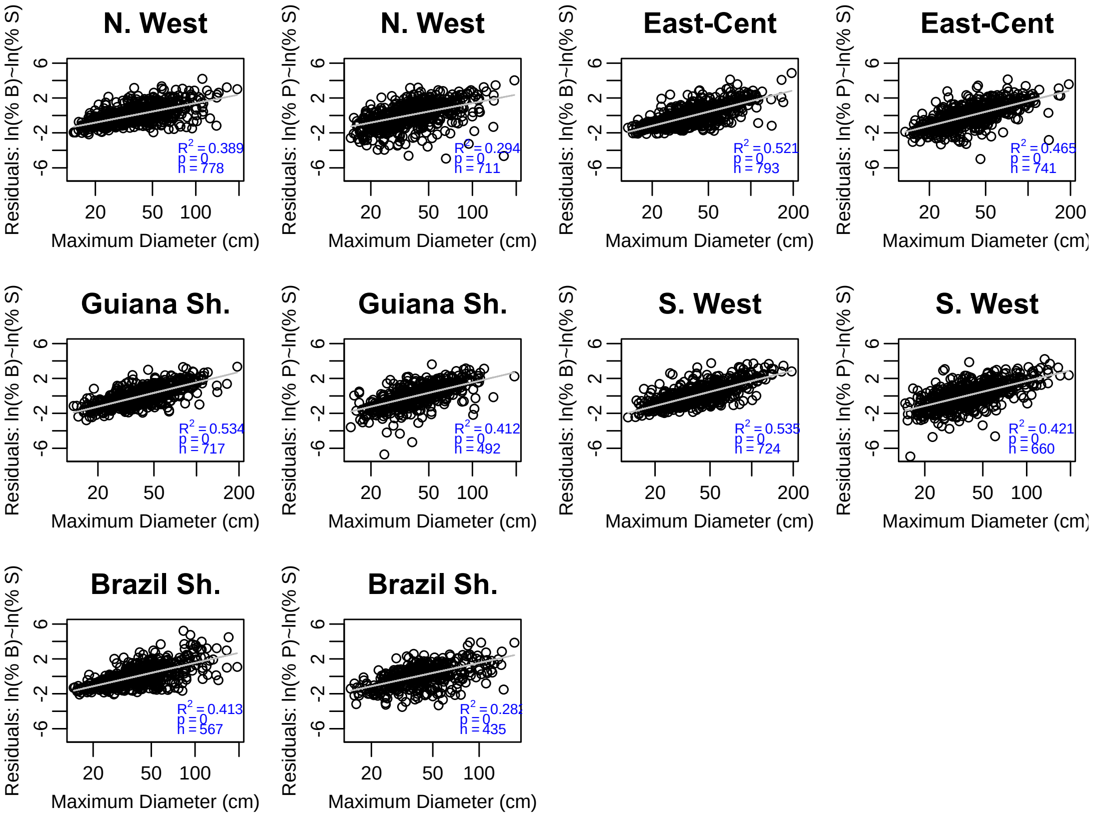

Supplementary Figure 6. Patterns of biomass and productivity contributions after controlling for stem abundance with maximum *D*. Relationships between the residuals from ln(% contribution to biomass)=a+b*ln(% contribution to stem number) and ln(% contribution to productivity)=a+b*ln(% contribution to stem number with maximum *D* for five Amazon regions. Regression models are plotted with grey lines.

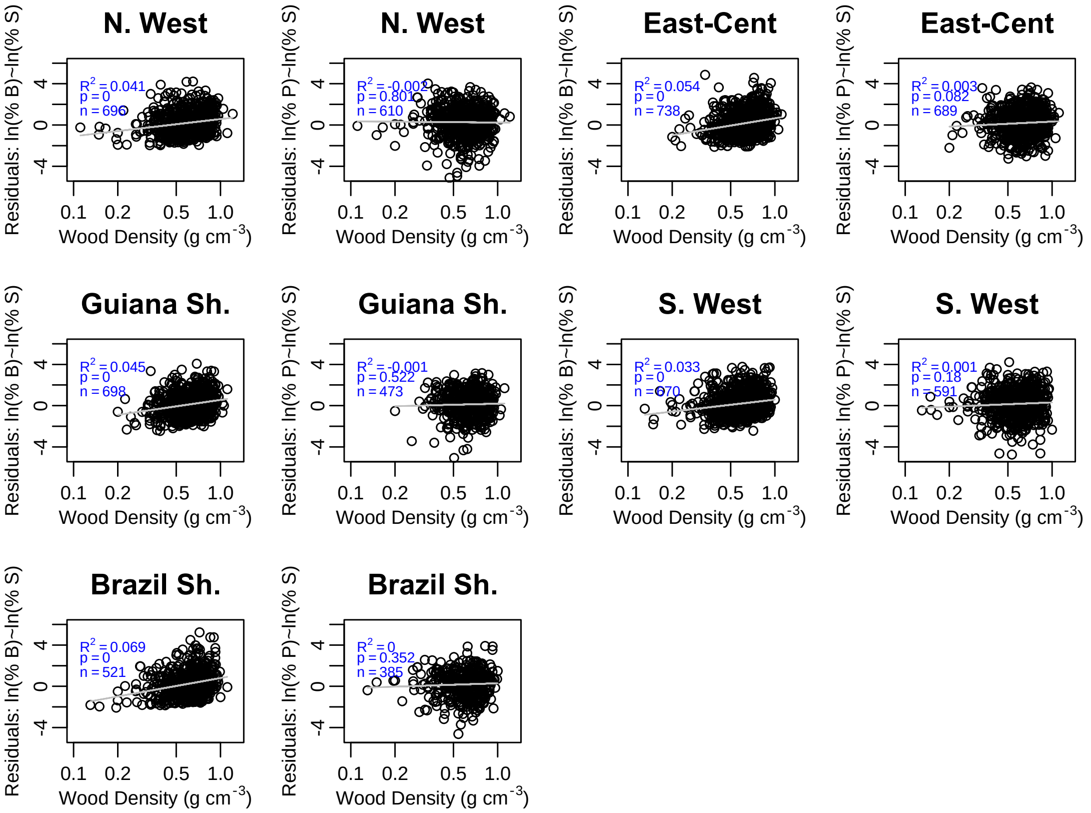

Supplementary Figure 7. Patterns of biomass and productivity contributions after controlling for stem abundance with wood density*.* Relationships between the residuals from ln(% contribution to biomass)=a+b*ln(% contribution to stem number) and ln(% contribution to productivity)=a+b*ln(% contribution to stem number with wood density for five Amazon regions. Regression models are plotted with grey lines.

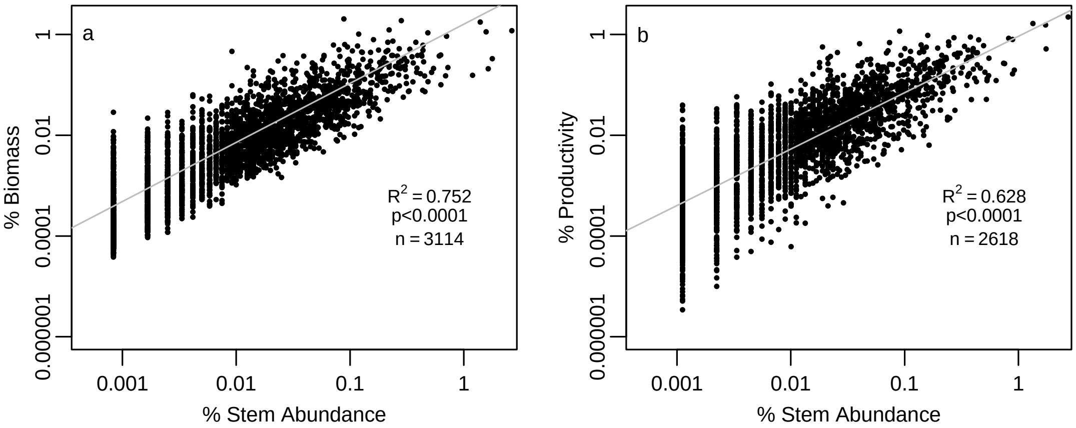

Supplementary Figure 8. Relationships between % contribution of species to stems and % contribution to (**a**) biomass and (**b**) productivity using data only from plots with at least 80 % of stems identified. Regression models are plotted with grey lines. Regression equation for % contribution to biomass: log(% biomass) = 0.20 + 1.17 log(% stem), regression equation for productivity: log(% biomass) = -0.04+ 1.12 log(% stem). All plots (326) are used for (**a**), and the reduced productivity dataset (148 plots) are used for (**b**). 77 species with negative or 0 productivity were excluded from (**b**). Plotted on log scale.

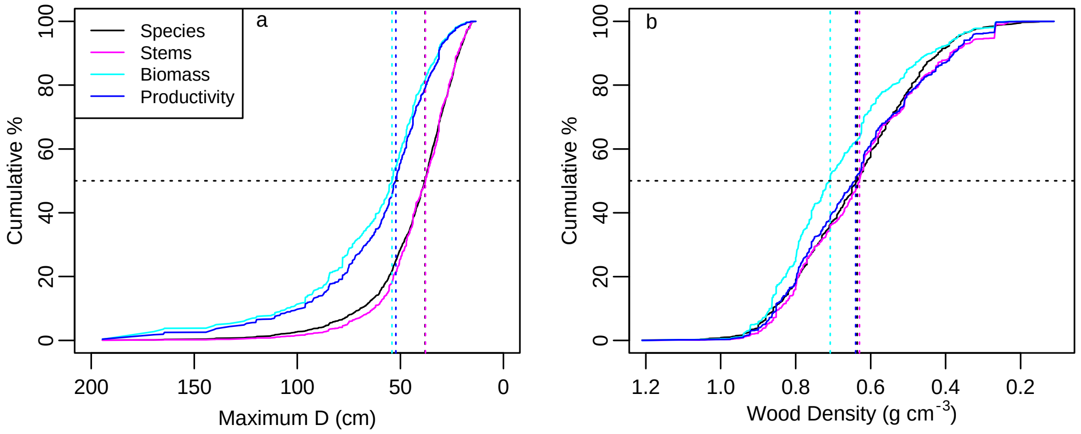

Supplementary Figure 9. Cumulative % contribution to species, stems, biomass and productivity against (**a**) maximum *D* and (**b**) wood density, using plots with at least 80 % of stems identified to species. Horizontal dashed black lines represent the mid-point of all metrics, vertical dashed lines show the trait value at the mid-point of each metric. All curves are based on the reduced productivity dataset, curves for biomass and stems are very similar when using the full dataset (data not shown).

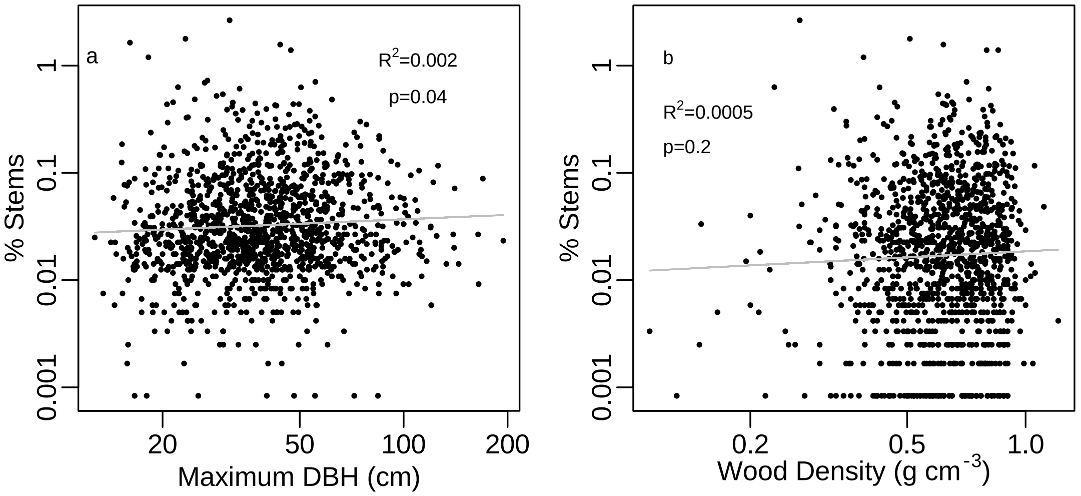

Supplementary Figure 10. Relationships between % contribution of species to stems with (**a**) maximum D (n = 1296) and (**b**) wood density (n = 1238), using plots with at least 80 % of stems identified to species. Regression models are plotted with grey lines. Plotted on log scale.

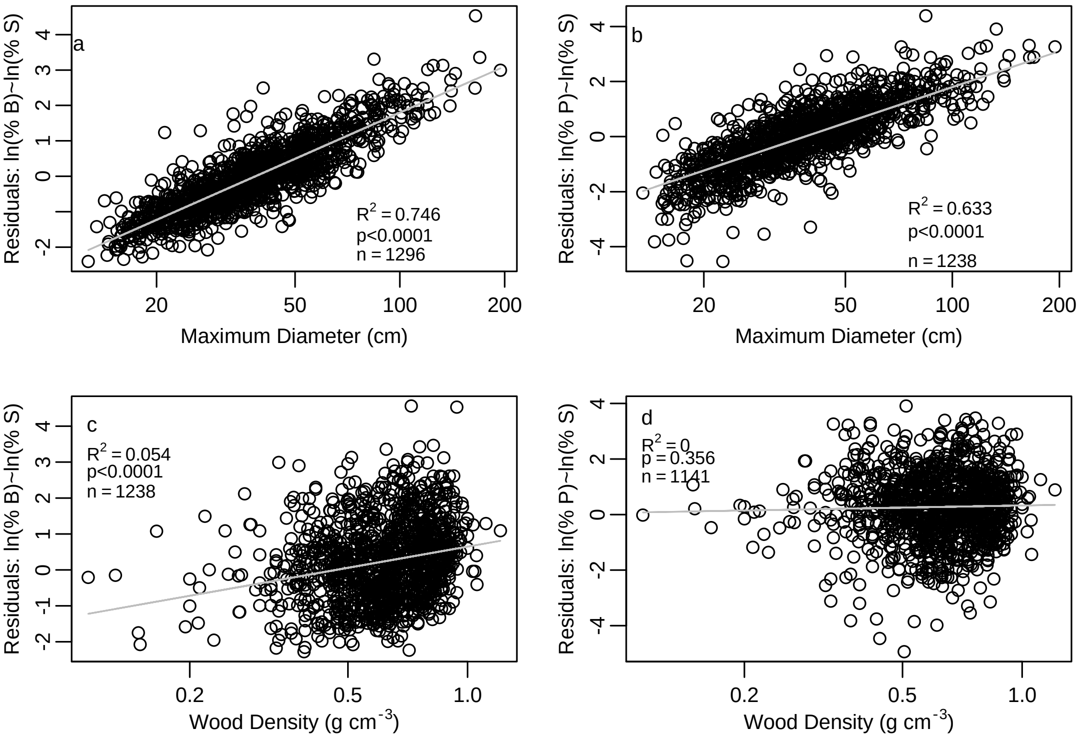

Supplementary Figure 11. Relationships between the residuals from ln(% contribution to biomass) = a + b * ln(% contribution to stem number) and maximum D (a) and wood density (c), relationships between the residuals from ln(% contribution to productivity) = a + b * ln(% contribution to stem number) with maximum D (b) and wood density (d). Regression models are plotted with grey lines. Maximum diameter and wood density plotted on a log scale.

Supplementary Tables

Supplementary Table 1. Top 20 species by stem number.

**Family**	**Species**	**Stems**	**% total stems**	**Cumulative % of total stems**
Arecaceae	*Iriartea deltoidea*	3814	1.85	1.85
Lecythidaceae	*Eschweilera coriacea*	3168	1.54	3.39
Arecaceae	*Astrocaryum murumuru*	2354	1.14	4.53
Moraceae	*Pseudolmedia laevis*	2243	1.09	5.62
Strelitziaceae	*Phenakospermum guyannense*	2032	0.99	6.60
Arecaceae	*Euterpe precatoria*	1991	0.97	7.57
Arecaceae	*Oenocarpus bataua*	1675	0.81	8.38
Fabaceae	*Eperua falcata*	1644	0.80	9.18
Arecaceae	*Euterpe oleracea*	1189	0.58	9.76
Burseraceae	*Tetragastris altissima*	1187	0.58	10.33
Arecaceae	*Socratea exorrhiza*	1129	0.55	10.88
Annonaceae	*Oxandra asbeckii*	1097	0.53	11.41
Lecythidaceae	*Lecythis persistens*	894	0.43	11.85
Burseraceae	*Protium hebetatum*	857	0.42	12.26
Violaceae	*Rinorea guianensis*	841	0.41	12.67
Myristicaceae	*Otoba parvifolia*	809	0.39	13.06
Chrysobalanaceae	*Licania alba*	808	0.39	13.45
Arecaceae	*Attalea butyracea*	802	0.39	13.84
Violaceae	*Leonia glycycarpa*	799	0.39	14.23
Myristicaceae	*Iryanthera juruensis*	785	0.38	14.61

Supplementary Table 2. Top 20 species by Productivity (reduced dataset).

Family	Species	Productivity (Mg/yr)	% total productivity	Cumulative % productivity
Arecaceae	*Iriartea deltoidea**	23.8	1.93	1.93
Lecythidaceae	*Eschweilera coriacea*	23.5	1.90	3.84
Moraceae	*Pseudolmedia laevis*	15.7	1.28	5.12
Lecythidaceae	*Bertholletia excelsa*	10.9	0.88	6.00
Fabaceae	*Vouacapoua americana*	10.3	0.84	6.84
Burseraceae	*Tetragastris altissima*	9.8	0.79	7.64
Arecaceae	*Attalea butyracea**	8.6	0.70	8.33
Fabaceae	*Eperua falcata*	8.1	0.65	8.99
Fabaceae	*Dicymbe altsonii*	8.0	0.65	9.64
Goupiaceae	*Goupia glabra*	7.9	0.64	10.28
Myristicaeae	*Otoba parvifolia*	7.7	0.62	10.90
Sapotaceae	*Manilkara huberi*	7.5	0.61	11.51
Lauraceae	*Chlorocardium rodiei*	7.4	0.60	12.12
Apocynaceae	*Aspidosperma excelsum*	7.3	0.59	12.71
Fabaceae	*Tachigali poeppigiana*	7.2	0.59	13.29
Fabaceae	*Dicorynia guianensis*	6.6	0.54	13.83
Fabaceae	*Inga alba*	6.4	0.52	14.35
Fabaceae	*Cedrelinga cateniformis*	6.2	0.51	14.86
Fabaceae	*Swartzia polyphylla*	6.1	0.50	15.36
Moraceae	*Brosimum rubescens*	5.9	0.48	15.84

* identifies monocot species for which productivity was estimated based on necromass production rather than biomass growth.

Supplementary Table 3. Contributions to total stems, biomass and productivity from largest and most densely wooded 50 % of species for five Amazon regions.

Region (Median Max *D* (cm); Median Wood Density (g cm^{-3}))		% contribution by largest 50 % of species	Maximum *D* at 50 % of metric (cm)	% contribution by 50 % most densely wooded species	Wood density at 50 % of metric (g cm^{-3})
North West (37.3; 0.61)	Stems	45.5	34.9	46.9	0.60
	Biomass	72.2	48.1	54.6	0.62
	Productivity	72.5	49.2	44.4	0.59
South West (39.3; 0.60)	Stems	44.8	36.3	39.4	0.55
	Biomass	78.5	55.4	50.5	0.61
	Productivity	75.6	51.7	44.3	0.59
Guiana Shield (42.6; 0.65)	Stems	52.6	44.0	69.8	0.75
	Biomass	84.9	62.0	82.4	0.79
	Productivity	84.4	62.0	74.9	0.76
East-Central (38.6; 0.67)	Stems	53.4	40.3	62.0	0.74
	Biomass	83.1	53.7	73.5	0.76
	Productivity	80.2	52.6	65.4	0.74
Brazilian Shield (40.7; 0.62)	Stems	42.0	36.5	59.6	0.63
	Biomass	79.2	55.5	71.8	0.69
	Productivity	73.9	53.5	63.7	0.65

Supplementary Table 4. Number of stem, biomass and productivity hyperdominant species using only plots with at least 80 % of stems identified to species.

	Full Dataset		Productivity Dataset		
	Stem Numbers	Biomass	Stem Numbers	Biomass	Productivity
Number hyperdominant species	194	138	186	124	141
% of species classed as hyperdominant	6.2	4.4	6.7	4.5	5.2
% accounted for by stem hyperdominants	50.0	44.0	50.0	44.0	42.0

The full dataset figures are based on 326 plots and productivity dataset figures are based on 148 multicensus plots.

Supplementary Table 5. Top 20 most abundant species by above ground woody biomass using only plots with at least 80 % of stems identified to species.

**Family**	**Species**	**Biomass**	**% total biomass**	**Cumulative % of biomass**	**Rank Stems**	**Rank Productivity**
Lecythidaceae	*Bertholletia excelsa*	1245	2.04	2.04	215	4
Lauraceae	*Chlorocardium rodiei*	1152	1.88	3.92	44	6
Lecythidaceae	*Eschweilera coriacea*	1076	1.76	5.69	5	2
Goupiaceae	*Goupia glabra*	752	1.23	6.92	62	19
Arecaceae	*Iriartea deltoidea*	726	1.19	8.10	1	1
Moraceae	*Pseudolmedia laevis*	688	1.13	9.24	4	3
Fabaceae	*Eperua falcata*	658	1.08	10.31	16	16
Fabaceae	*Dicymbe altsonii*	623	1.02	11.33	143	5
Burseraceae	*Tetragastris altissima*	562	0.92	12.25	8	9
Elaeocarpaceae	*Sloanea guianensis*	483	0.79	13.05	100	28
Vochysiaceae	*Qualea paraensis*	452	0.74	13.79	55	14
Apocynaceae	*Aspidosperma excelsum*	428	0.70	14.49	66	11
Moraceae	*Brosimum rubescens*	428	0.70	15.19	28	21
Sapotaceae	*Manilkara huberi*	387	0.63	15.82	207	22
Fabaceae	*Cedrelinga cateniformis*	379	0.62	16.44	281	12
Arecaceae	*Attalea phalerata*	374	0.61	17.05	20	36
Eupohorbiaceae	*Hura crepitans*	361	0.59	17.65	152	32
Moraceae	*Brosimum alicastrum*	346	0.57	18.21	193	39
Lecythidaceae	*Eschweilera sagotiana*	319	0.52	18.73	48	43
Olacaceae	*Minquartia guianensis*	311	0.51	19.24	32	42

Productivity ranks are based on the 148 plot productivity dataset.

Supplementary Table 6. Top 20 species by stem number using only plots with at least 80 % of stems identified to species.

**Family**	**Species**	**Stems**	**% total stems**	**Cumulative % of total stems**
Arecaceae	*Iriartea deltoidea*	3178	2.65	2.64
Arecaceae	*Astrocaryum murumuru*	2138	1.78	4.43
Strelitiziaceae	*Phenakospermum guyannense*	1968	1.64	6.07
Moraceae	*Pseudolmedia laevis*	1886	1.57	7.64
Lecythidaceae	*Eschweilera coriacea*	1676	1.40	9.03
Arecaceae	*Euterpe precatoria*	1434	1.19	10.23
Arecaceae	*Oenocarpus bataua*	871	0.72	10.95
Burseraceae	*Tetragastris altissima*	848	0.71	11.66
Burseraceae	*Protium hebetatum*	832	0.69	12.35
Arecaceae	*Socratea exorrhiza*	756	0.63	12.98
Myristicaeae	*Otoba parvifolia*	753	0.63	13.61
Lecythidaceae	*Eschweilera wachenheimii*	731	0.61	14.22
Violaceae	*Leonia glycycarpa*	650	0.54	14.76
Myristicaeae	*Iryanthera juruensis*	626	0.52	15.28
Euphorbiaceae	*Sagotia brachysepala*	582	0.48	15.76
Fabaceae	*Eperua falcata*	580	0.48	16.25
Arecaceae	*Oenocarpus bacaba*	547	0.46	16.70
Malvaceae	*Quararibea wittii*	543	0.45	17.15
Cannabaceae	*Celtis schippii*	533	0.44	17.60
Arecaceae	*Attalea phalerata*	526	0.44	18.04

Supplementary Table 7. Top 20 species by above ground woody productivity using only plots with at least 80 % of stems identified to species.

**Family**	**Species**	**Productivity**	**% total Productivity**	**Cumulative % of productivity**
Arecaceae	*Iriartea deltoidea**	18.60	2.23	2.23
Lecythidaceae	*Eschweilera coriacea*	13.77	1.65	3.88
Moraceae	*Pseudolmedia laevis*	12.86	1.54	5.43
Lecythidaceae	*Bertholletia excelsa*	9.72	1.17	6.59
Fabaceae	*Dicymbe altsonii*	8.00	0.96	7.55
Lauraceae	*Chlorocardium rodiei*	7.44	0.89	8.44
Fabaceae	*Tachigali poeppigiana*	7.21	0.86	9.31
Myristaceae	*Otoba parvifolia*	7.01	0.84	10.15
Burseraceae	*Tetragastris altissima*	6.64	0.80	10.95
Arecaceae	*Attalea butyracea**	6.55	0.79	11.73
Apocynaceae	*Aspidosperma excelsum*	5.92	0.71	12.44
Fabaceae	*Cedrelinga cateniformis*	5.77	0.69	13.13
Moraceae	*Ficus coerulescens*	5.48	0.66	13.79
Vochysiaceae	*Qualea paraensis*	5.19	0.62	14.41
Fabaceae	*Anadenanthera colubrina*	5.05	0.60	15.02
Fabaceae	*Eperua falcata*	5.01	0.60	15.62
Moraceae	*Poulsenia armata*	4.87	0.58	16.20
Meliaceae	*Swietenia macrophylla*	4.72	0.57	16.77
Goupiaceae	*Goupia glabra*	4.67	0.56	17.33
Moraceae	*Clarisia racemosa*	4.48	0.54	17.87

* shows palm species where productivity estimates are based on necromass production.

Supplementary Table 8. Contributions to total stems, biomass and productivity from largest and most densely wooded 50 % of species using only plots with at least 80 % of stems identified to species.

	% contribution by largest 50 % of species	Maximum D^a at 50 % of metric (cm)	% contribution by densest 50 % of species	Wood density[b] at 50 % of metric (g cm^{-3})
Stems	49.1	37.8	48.0	0.630
Biomass	81.5	54.1	62.7	0.708
Productivity	78.9	52.2	51.7	0.640

[a] Median Max DBH species 38.0 cm

[b] Median WD species: 0.636 g cm^{-3}
